# Allergic bronchopulmonary aspergillosis with coexistant aspergilloma: a case report

**DOI:** 10.1186/1752-1947-4-309

**Published:** 2010-09-20

**Authors:** Izidor Kern, Anton Lopert

**Affiliations:** 1 Laboratory of Pathology, University Clinic of Respiratory and Allergic Diseases, Golnik, Slovenia; 2 Private Practice for Pulmonary and Allergic Diseases, Murska Sobota, Slovenia

## Abstract

**Introduction:**

The coexistence of allergic bronchopulmonary aspergillosis and aspergilloma is rare.

**Case presentation:**

We present the case of a 56-year-old Caucasian man who worked as a farmer, with infiltrates in the right lower and middle lung lobes, partial consolidation of the middle lobe and with previous diagnosis of chronic obstructive bronchitis. Evaluation of our patient led to the diagnosis of allergic bronchopulmonary aspergillosis with coexistent aspergilloma in the right lower lobe. He was treated with oral methylprednisolone and itraconazole. At the five-year follow-up he is without any sign of recurrence.

**Conclusion:**

Aspergillus infection after the inhalation of spores in the form of a hypersensitivity reaction and saprophytic colonization can be coexistent.

## Introduction

Allergic bronchopulmonary aspergillosis (ABPA) is a complex hypersensitivity reaction in patients with asthma, which occurs when bronchi are colonized by the fungus *Aspergillus*, most often *Aspergillus fumigatus*. The disease is characterized by type I and type III hypersensitivity reactions. The incidence may be as high as 6% of patients with asthma [1]. Repeated episodes of bronchial obstruction, inflammation and mucoid impaction can lead to bronchiectasis, fibrosis and respiratory compromise.

Aspergilloma is a saprophytic growth of fungus, usually *A. fumigatus*, in the lumen of an existing cavity, which does not invade the tissue. Fungus ball formation has been observed in an old tuberculosis cavity, bronchiectasis, abscess or in a congenital cyst.

We present a case of the coexistence of ABPA and aspergilloma. A patient with ABPA developed a cavitary pulmonary lesion with characteristic radiological appearances of aspergilloma. The management and time sequence of the ABPA and aspergilloma occurrences are discussed.

## Case presentation

A 56-year-old Caucasian Slovenian man, who worked as a farmer and was a non-smoker, was treated for bronchopneumonia in the right upper lobe in 1970 and 1974. The infiltrate resolved after antibiotic therapy with penicillin. He also had symptoms of chronic bronchitis, but he did not require therapy.

In 1996, our patient presented with persistent cough, dyspnea and sweats. He had no fever. A general practitioner prescribed him a macrolide antibiotic, but there was no response to this treatment and our patient was referred to a pneumologist. A chest radiograph showed an infiltrate with cavitation in the right lower lobe. Sputum testing for tuberculosis was negative. Tuberculine testing was positive and our patient received tuberculostatic therapy without any improvement over the following four weeks.

**Figure 1:**
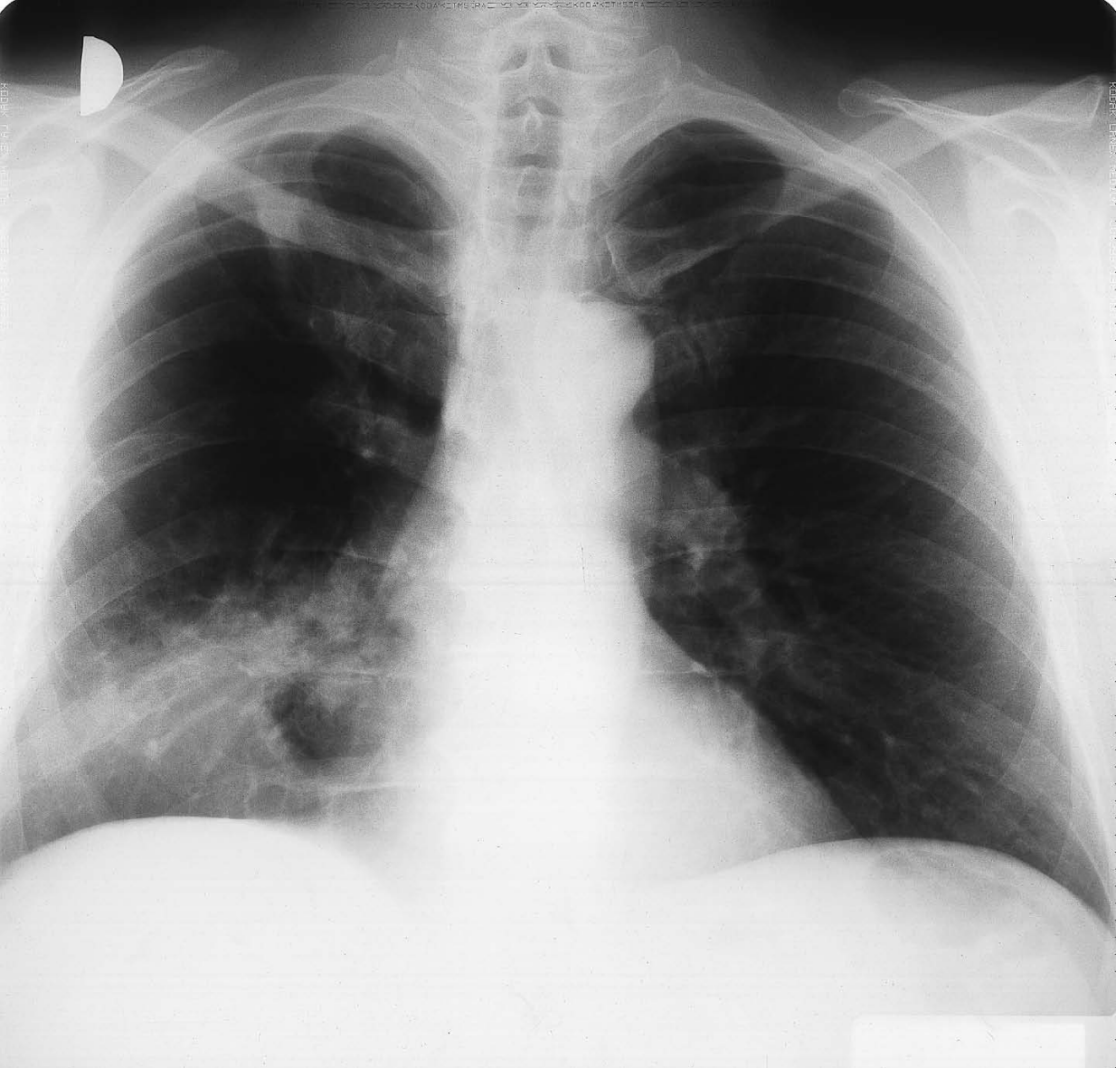
**Chest radiograph showing an infiltrate with a cavitation in the right lower and middle lobes**.

**Figure 2:**
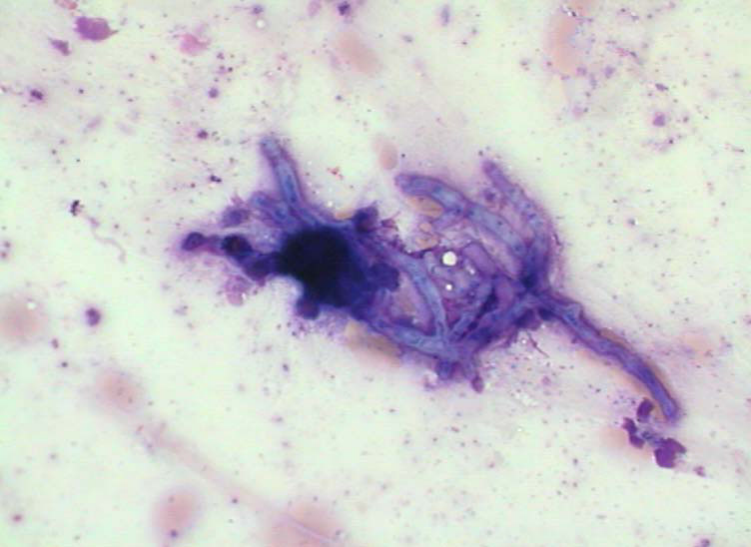
**Fungal hyphae morphologically corresponding to *Aspergillus *sp. May Grunwald-Giemsa (MGG) staining, original magnification × 400**.

On admission to our hospital, he was afebrile and eupnoic. No lymphadenopathy was found. At the auscultation, prolonged expiration and weaker lung sounds over the right basal field were heard. A chest radiograph showed the infiltrates with cavitation in the right lower lobe and in the middle lobe with consolidation of the latter (Figure 1). Laboratory data showed an elevated erythrocyte sedimentation rate of 66 mm/h and a normal white cell count. *Pseudomonas aeruginosa *was isolated from the sputum and our patient was treated with ciprofloxacin. Pulmonary function tests showed reduced forced vital capacity (FVC) of 2900 mL (-36%), reduced forced expiratory volume in the first second (FEV_1_) of 1900 mL (-40%), with the Tiffeneau index of 65%. Arterial blood gas analysis showed only mild hypoxemia (pO_2 _9.5 kPa). We also performed fiberbronchoscopy and found a necrotic and purulent mass in the middle lobe bronchus that had practically closed the lumen. Cytological examination of the material obtained with bronchial brushing disclosed numerous eosinophils, Charcot-Leyden crystals and fungal structures morphologically corresponding to *Aspergillus *spp. (Figure 2). There were no malignant cells. Histological examination of the bronchial biopsy specimen showed chronic inflammatory changes in bronchial mucosa with mononuclear cells and focally granulocytes infiltration. Control fiberbronchoscopy after one week revealed inflammatory infiltration of mucosa in the middle lobe bronchus. Histopathology showed non-specific inflammatory changes of bronchial mucosa. Cutaneous testing for the *A. fumigatus *antigen was strongly positive. Total serum immunoglobulin E (IgE) concentration was elevated (505 IU/mL), specific IgE against *A. fumigatus *were strongly elevated (27.9 IU/mL). Serum precipitating antibodies were elevated, specific IgG against *A. fumigatus *(55 IU/mL) and against *A. flavus *(51 IU/mL). Metacholine bronchoprovocation testing showed bronchial hyper-responsiveness with the reversibility of obstruction after the bronchodilator. Our patient received methylprednisolone, 32 mg daily. The dose was gradually tapered during the subsequent months.

**Figure 3:**
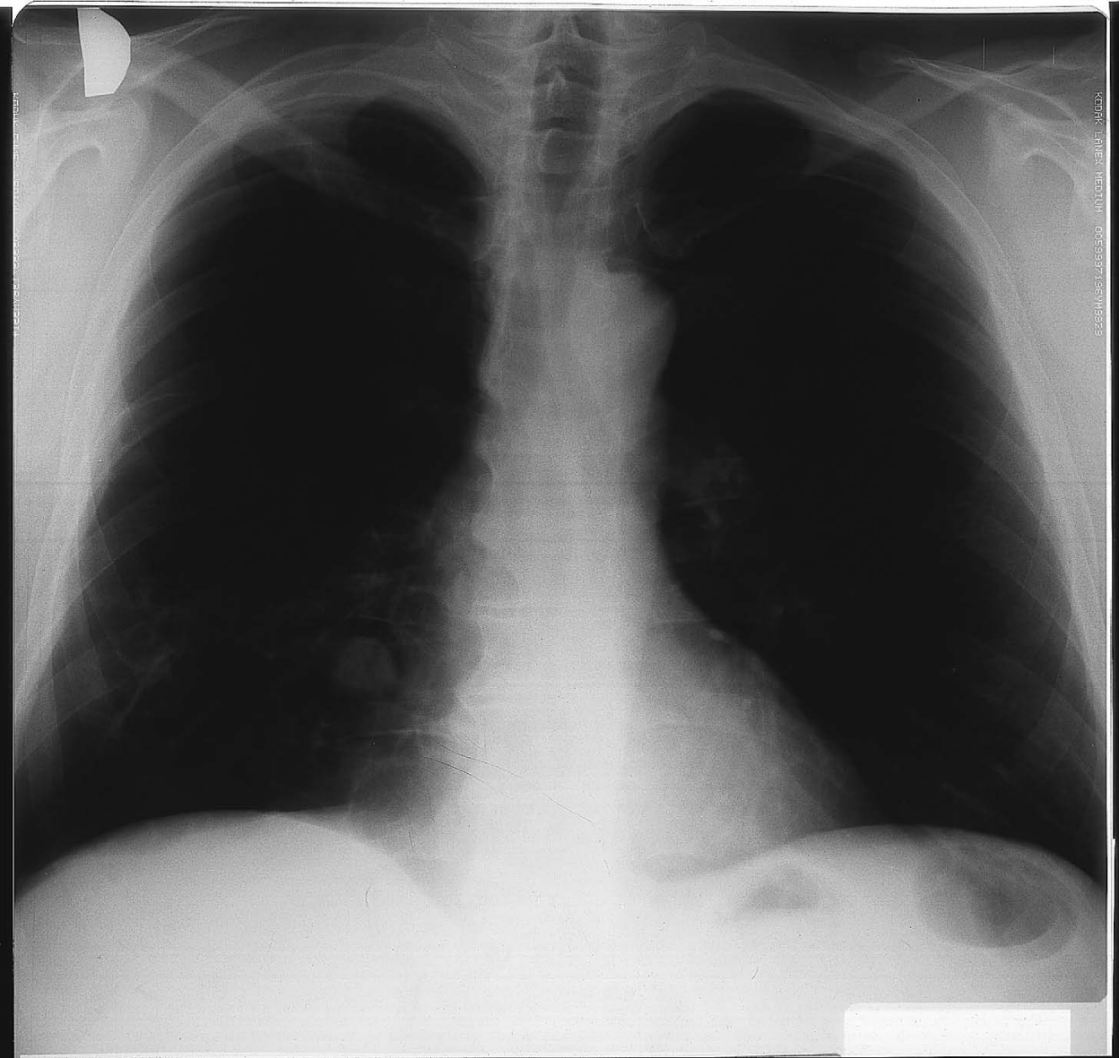
**A cavitary lesion with air crescent in the right lower lobe seen on chest radiograph is characteristic of an aspergilloma**.

Pulmonary function tests improved: FVC from 2900 mL to 3100 mL and FEV_1 _from 1900 mL to 2200 mL. Total serum IgE concentration fell to 186 IU/ml, specific IgE against *A. fumigatus *to 6.1 IU/ml, specific IgG precipitating antibodies against *A. fumigatus *and *A. flavus *fell to 30 IU/mL and 32 IU/mL, respectively. The chest radiograph disclosed that the infiltrate in the middle lobe resolved, but in the posterior segment of the right lower lobe a cavitary pulmonary lesion with the diameter of 3 cm and with an air crescent was formed. The radiological appearances of this lesion were characteristic of an aspergilloma (Figure 3). Itraconazole 200 mg daily for two months was added to methylprednisolone.

**Figure 4:**
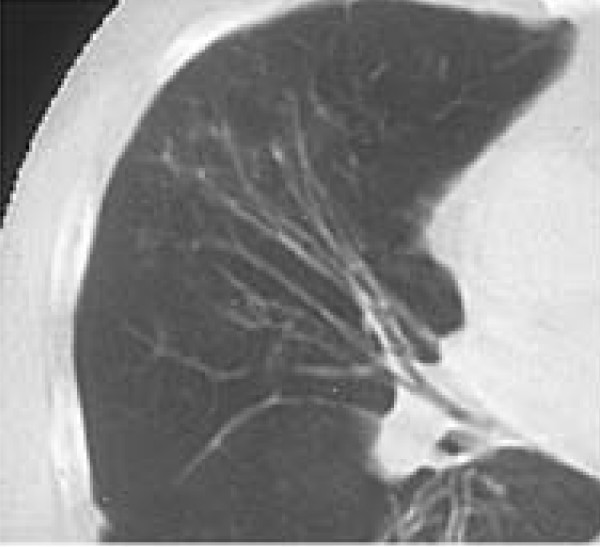
**Computed tomography revealed tram-line shadows and cylindrical bronchiectasis in the middle lobe**.

Subsequent controls showed further clinical improvement. The chest radiographs and chest computed tomography (CT) showed that the aspergilloma became smaller, measuring 2 cm in diameter, and was partially calcified. Fibrotic changes in the right lower and middle lobes were present. Chest CT also showed tram-line shadows of bronchial wall thickening and cylindrical bronchiectasis in the middle lobe (Figure 4). Specific IgE against *A. fumigatus *remained elevated (9.4 IU/mL) while specific IgG precipitating antibodies against *A. fumigatus *and *A. flavus *normalized (7 IU/mL and 19 IU/mL, respectively). Our patient receives inhalatory steroid and short-acting β_2_-agonist. He is being followed over a five-year period with no signs of recurrence.

## Discussion

The coexistence of an aspergilloma and ABPA is a rare finding. Some authors described a development of aspergilloma secondary to ABPA [2-4], and others reported ABPA consequent to aspergilloma [5,6]. In 1973 Safirstein described an occurrence of aspergilloma consequent to ABPA [4]. Israel (1980) reported the rapid development of aspergilloma secondary to ABPA with a brief history of preceding asthma [5]. Shah reported, in 1989, two patients with ABPA who later developed aspergillomas and were followed over 30 and 18 months, respectively [6]. In 1979, Ein *et al*. reported two patients with an ABPA-like syndrome consequent to aspergilloma with subjective and objective improvements after the administration of corticosteroid therapy [7]. Rosenberg (1984) described a patient with such a combination followed over a two-year period [8]. Our patient has been followed over five years and has not shown signs of recurrence which can occur after many years of remission [9].

In 1991, Hefti described a patient with ABPA and aspergilloma in the left upper lobe following a chronic abscessed pneumonia [10]. Shah reported a patient with ABPA and with a middle lobe syndrome [11]. Our patient had some symptoms of obstructive pulmonary disease as early as in 1970, but his dyspnea was not severe and he did not take any therapy. After undergoing bronchopneumonia in 1970 and 1974, only small fibrotic changes were seen on chest radiographs of the right upper lobe of the lung. There was no pathology in the middle or right lower lobes. Thus, at that time, no pulmonary cavities were described where aspergilloma formation could grow. Pulmonary tuberculosis was never proven. Nevertheless, on admission into our hospital in August 1996, a cavity was suggested inside the infiltrate in the right lower lung (Figure 1), which was not seen on the chest radiographs from 1970. It is possible that in 26 years, a cavitary lesion developed where aspergilloma later occurred. The patient had a pulmonary infiltrate in the right lower lobe with endobronchial obstruction of the middle lobe bronchus by an impacted mucus plug. Later, a CT-scan showed cylindrical bronchiectasis of the middle lobe and aspergilloma in the right lower lobe.

This patient was a farmer and therefore his exposure to an environment rich in the *Aspergillus *spores was high. Vernon *et al*. found that patients with ABPA had not been more exposed to potentially rich sources of *A. fumigatus *than atopic control patients. Specific host susceptibility seems to be more important in the pathogenesis of ABPA than environmental factors. However, once the patient is sensitized a minor increase in spore concentration can cause symptomatic disease [12]. The presence of the type I hypersensitivity described in some patients with aspergilloma suggests an immunologic component to this disease which could contribute to a chronic inflammatory response to *Aspergillus*.

Oral corticosteroids are the therapy of choice in ABPA. Itraconazole may have its role in therapy, especially in cases where oral corticosteroids are contraindicated. In patients requiring high doses of oral steroids itraconazole may allow a reduction in dose, but should not replace the need of corticosteroid treatment [1]. Corticosteroid treatment should result in the reduction of the IgE level (remission or stage II disease). Resolution of pulmonary infiltrates and clinical improvement are generally accompanied by at least a 35% reduction in serum total IgE, which was seen also in our patient. He was treated with oral methylprednisolone for four months. Itraconazole 200 mg daily was added for two months after the resolution of the infiltrate when the aspergilloma in the right lower lobe became visible.

## Conclusions

We conclude that in a patient with ABPA who develops a cavitary lesion, aspergilloma should be considered. The time sequence of ABPA and aspergilloma occurrence can also be reversed. After corticosteroid and itraconazole treatment both subjective and objective improvements are experienced.

## Consent

Written informed consent was obtained from the patient for the publication of this case report and any accompanying images. A copy of the written consent is available for review by the Editor-in-Chief of this journal.

## Competing interests

The authors declare that they have no competing interests.

## Authors' contributions

AL analyzed and interpreted the patient data. IK performed the pathological examination and was a major contributor in writing the manuscript. All authors read and approved the final manuscript.
